# The effect of elastic and viscous force fields on bimanual coordination

**DOI:** 10.1007/s00221-023-06589-7

**Published:** 2023-03-14

**Authors:** Jaskanwaljeet Kaur, Shannon Proksch, Ramesh Balasubramaniam

**Affiliations:** 1 Sensorimotor Neuroscience Laboratory, Cognitive and Information Sciences, University of California, 5200 N Lake Road Merced, Merced, CA 95343 USA; 2 Department of Psychology, Augustana University, Sioux Falls, SD 57197 USA

**Keywords:** HKB model, Kinarm robotic exoskeleton, Force fields, In-phase coordination, Anti-phase coordination

## Abstract

Bimanual in-phase and anti-phase coordination modes represent two basic movement patterns with distinct characteristics—homologous muscle contraction and non-homologous muscle contraction, respectively. A method to understand the contribution of each limb to the overall coordination pattern involves detuning (Δω) the natural eigenfrequency of each limb. In the present experiment, we experimentally broke the symmetry between the two upper limbs by adding elastic and viscous force fields using a Kinarm robot exoskeleton. We measured the effect of this symmetry breaking on coordination stability as participants performed bimanual in-phase and anti-phase movements using their left and right hand in 1:1 frequency locking mode. Differences between uncoupled frequencies were manipulated via the application of viscous & elastic force fields and using fast and slow oscillation frequencies with a custom task developed using the Kinarm robotic exoskeleton. The effects of manipulating the asymmetry between the limbs were measured through the mean and variability of relative phase (*ϕ*) from the intended modes of 0 ° or 180 °. In general, participants deviated less from intended phase irrespective of coordination mode in all matched conditions, except for when elastic loads are applied to both arms in the anti-phase coordination. Second, we found that when force fields were mismatched participants exhibited a larger deviation from the intended phase. Overall, there was increased phase deviation during anti-phase coordination. Finally, participants exhibited higher variability in relative phase in mismatched force conditions compared to matched force conditions, with overall higher variability during anti-phase coordination mode. We extend previous research by demonstrating that symmetry breaking caused by force differences between the limbs disrupts stability in each coordination mode.

**Supplementary Information:**

The online version contains supplementary material available at 10.1007/s00221-023-06589-7.

## Introduction

The ability to coordinate both upper limbs is an essential aspect of human movement. Being able to coordinate enables us to perform daily tasks like walking, eating, typing, playing an instrument, or dancing. Loss of this essential ability significantly affects quality of life and has been studied extensively in patients who have suffered a stroke (Bansil et al. 2012) have Parkinson’s disease (Mazzoni et al. 2012) or have sustained a traumatic brain injury (TBI) causing motor problems (O’Suilleabhain and Dewey 2004). When performing bimanual movements, young, healthy individuals perform these activities with ease, whereas this ability to coordinate with precision declines as individuals age (Morrison and Newell 2012). But even within healthy individuals, the amount of effort it takes to perform bimanual activities varies as coordination patterns change.

One way to measure and understand changes in rhythmic bimanual coordination patterns is to investigate two possible movement patterns that have been extensively studied: in-phase coordination and anti-phase coordination (Kelso 1984). During the in-phase coordination mode, both hands move simultaneously in a mirror-symmetrical pattern in line with the body’s midline thereby recruiting homologous muscle groups, whereas during anti-phase coordination mode, both hands move simultaneously in the opposite directions, thereby recruiting non-homologous muscle groups (Swinnen 2002). In early work performed by Kelso and colleagues, rhythmic in-phase movements were consistently found to be easier to perform (i.e., lower attentional load), while rhythmic anti-phase movements take more practice to perform (i.e., higher attentional load) (Wuyts et al. 1996; Monno et al. 2002; Ridderikhoff et al. 2008). Additionally, when performing the anti-phase movement, increasing the frequency of movement (speed) causes the anti-phase movement to phase transition to the in-phase movement pattern–the more stable of the two coordination modes (Haken et al. 1985). The converse transition (from in-phase to anti-phase coordination mode) rarely occurs (Franz et al. 1991), and is commonly referred to as a form of hysteresis (Serrien et al. 2018). Prior experiments with hand-held pendulums have shown similar trends, whereby the in-phase movement is stable compared to the anti-phase movement and phase transition occurs when the movement frequency is increased (Riley et al. 2001; Temprado et al. 2007).

Previous studies have quantified interlimb performance during rhythmic coordination between two limbs by considering the mean and standard deviation of relative phase ϕ, defined as the phase difference between two oscillating segments (i.e., hands) (Semjen et al. 1995; Swinnen et al. 1996; Debaere et al. 2004). The in-phase and anti-phase movement can be formalized using the following Haken-Kelso-Bunz (HKB) model of coordination, Eq. 1

1 $$V\left( \phi \right) = - a\cos \left( \phi \right) - b {\text{cos}}\left( {2\phi } \right)$$

where, *V* is defined as the potential function and is the ratio representing relative stability of the in-phase and anti-phase movement patterns. The HKB model has since been expanded upon to add a detuning or symmetry breaking term (∆ω), seen in Eq. 2 (Fuchs and Jirsa 2000). The ∆ω term is used for oscillators with different eigenfrequencies, i.e., different inherent or preferred movement frequencies (Bressler & Kelso 2001, 2016; Fuchs and Kelso 1994; Park and Turvey 2008; Peper et al. 2004).

2 $$V\left( \phi \right) = - \Delta \omega \phi - \alpha \cos \left( \phi \right) - b\cos \left( {2\phi } \right)$$

The ∆ω term can be quantified by looking at the difference between relative phase relationships that are produced and comparing them to the intended coordination modes. For instance, smaller differences in the eigenfrequencies cause small shifts in movement patterns, whereas larger differences in eigenfrequencies cause larger shifts in movement patterns. Based on this, it may be argued that manipulating the ∆ω term by applying perturbations, so as to alter the eigenfrequency (or the natural frequency) of movement could help us understand how bimanual coordination is affected during in-phase and anti-phase coordination modes in a steady-state system coordination dynamic. Here, we developed a novel task using the Kinarm robotic exoskeleton (BKIN Technologies Ltd, Ontario, Canada), a device which has a high temporal resolution (1000 Hz) and a spatial resolution in the millimeter range to precisely assess upper limb bimanual coordination. Based on prior literature (Sternad et al. 1995; Amazeen et al. 1995; Riley et al. 2001), we hypothesized that breaking the symmetry between the upper limbs will affect coordination stability and variability between the hands during bimanual coordination, specifically, (1) applying matched perturbations (applying same load on both arms) will result in lower phase deviation from the intended phase and lower variability, whereas, (2) applying mismatched perturbations (applying varying loads on both arms) will result in higher deviation from the intended relative phase and increased variability. The overall objective of the present experiment was to understand how perturbing the motor system, by way of applying velocity (viscous) and position (elastic) dependent forces, would influence coordination stability.

## Methods

### Participants

Thirty seven healthy participants participated in the study. Of the thirty seven, data were excluded from four participants who either did not complete the study or did not follow instructions of the task. Thirty-three participants’ data were thus analyzed (age: 21.69 ± 2.54, 15 male). For eight of the thirty-three participants the data for one of the load conditions was not collected due to an oversight in stimulus presentation. The remaining twenty-five participants completed all load conditions. The experiment was approved by the Institutional Review Board of the University of California, Merced and was performed in agreement with the Declaration of Helsinki. All participants provided written informed consent prior to joining the experiment.

### Handedness measurements

Handedness was assessed by asking participants to complete the 4-item Edinburgh Handedness Inventory (EHI) – Short Form (Veale 2014). The EHI accesses hand dominance in daily activities (e.g., writing, throwing). The laterality quotient (LQ) of hand dominance ranges from  – 100 (left-handed) to 100 (right-handed): an LQ between  – 100 &  – 61,  – 60 & 60, and 61 and 100 were considered left handers, mixed handers, and right handers, respectively. In the present study 87% of the participants were right-handed (*N* = 29) and 12% of the participants were left-handed (*N* = 4).

### Experimental device

**Fig. 1:**
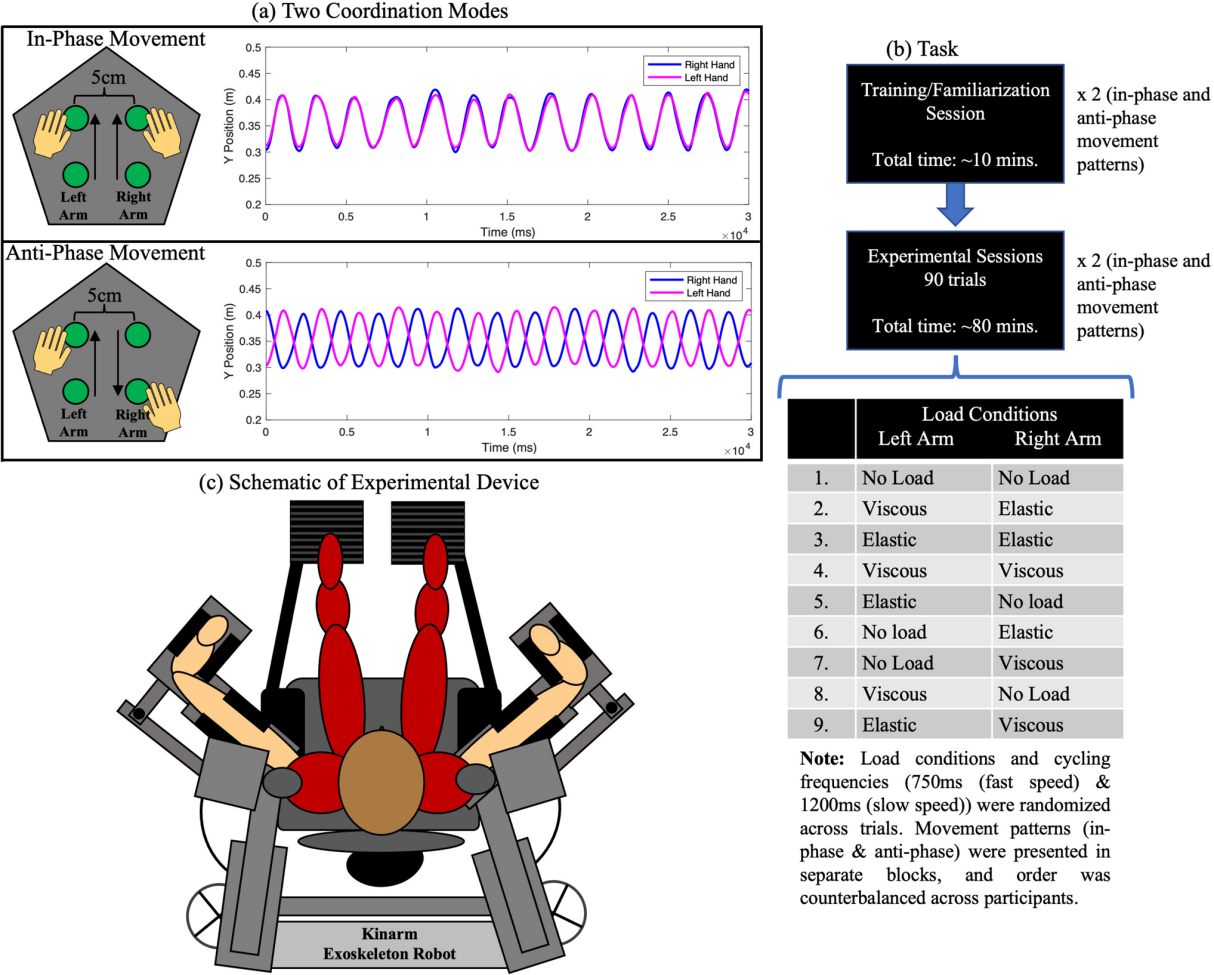
Experimental Setup. **a** Schematic of the two coordination modes (In-phase and Anti-phase). The waveforms depict one trials of in-phase and anti-phase movement, respectively. **b** Task design depicts the order of events during the task for both the in-phase and anti-phase coordination modes: a training/familiarization session was completed, followed by 90 randomized trials consisting of 9 different load conditions across 750 and 1200 ms cycling frequencies. **c** Kinarm Upper limb Robotic Exoskeleton

The experiment was performed using the Kinarm upper limb robotic exoskeleton. The Kinarm exoskeleton has been utilized in primate studies, human research studies, along with clinical assessments to diagnose stroke and other motor deficits (Bansil et al. 2012). At a sampling rate of up to 1000 Hz, it is able to accurately and precisely quantify motor and sensory characteristics of both healthy and neurologically disabled participants (Kenzie et al. 2014; Dukelow et al. 2010). The Kinarm device comprises a height-adjustable chair with bilateral arm and hand support platforms, with a monitor linked to the operator’s computer and a screen underneath the monitor to display the task being performed (task schematic in Fig. 1a). This environment allows participants to perform two-dimensional movements while being able to observe and interact with the stimuli being projected onto the screen, enabling both the visual stimuli and the ability to perform bimanual arm movements to be conducted within the same workspace. As participants interact with stimuli, perturbations or force fields can be applied so as to interfere with the planned movement in order to understand the impact of their perturbations of motor movements (Brown et al. 2007). Participants’ movements were continuously recorded via the Dexterit-E software during the task performance at a sampling rate of 1000 Hz (3.8v, BKIN Technologies Ltd, Ontario, Canada). Once the experiment was completed, the recorded data was then automatically saved as a c3d data file, containing the hand position coordinates (x, y), and movement velocity and acceleration of the arm along the transverse plane.

### In-phase/anti-phase coordination modes

We developed a bimanual coordination task on Simulink (R2015a, The MathWorks, USA) and Dexterit-E to probe how perturbing the motor systems by applying force fields, i.e., viscous and/or elastic forces, affects in-phase and anti-phase movements in a bimanual coordination task. The viscous force is a velocity-dependent force that acts like a dampener or friction applied to the limb, such that an increase in velocity was followed by an increase in resistance. The elastic force is a position-dependent force where the lateral force scaled with hand position relative to the start position. The force that the robot applied to the hand was always orthogonal to the direction of movement and can be seen as follows:

3 $$F = k\left[ {\begin{array}{*{20}c} 0 & { - 1} \\ 1 & 0 \\ \end{array} } \right]\left[ {\begin{array}{*{20}c} {\dot{x}} \\ {\dot{y}} \\ \end{array} } \right]$$

where *F* is the vector of the forces in the horizontal plane, ẋ and ẏ represent the hand velocities in the horizontal place, and k indicates the viscosity of the force field. In the viscous (velocity-dependent force field) the force applied is *k* =  – 15N/m, whereas the elastic (position-dependent force field) the force applied is *k* = 15N/m.

As can be seen in Fig. 1a, the four target circles were displayed 5 cm from one another. The target size and distance were determined by piloting the experiment with adult (> 18 years) participants. For both the in-phase and anti-phase coordination modes, the experimental setup was similar, but the movements differed depending on the coordination mode being tested. Prior to starting the task, participants were trained on the coordination mode they were randomized to start with (in-phase or anti-phase). They moved their hands to white targets (initial starting position), which caused red targets to start flashing five times (red targets flashed in the same location as the green targets seen in Fig. 1a) cueing the participants regarding the required cycling frequency. The cycling frequencies used for this task was 750 ms (fast speed) and 1200 ms (slow speed). The cycling frequencies were selected based on the pilot experiment with adults, where the goal was to provide a comfortable speed to perform rhythmic movements without the potential of phase transitioning during each movement.

There was a total of 9 load conditions, which were classified into four main movement patterns (Fig. 1b): in-phase coordination mode at 750 ms cycling frequency (fast speed), in-phase coordination mode at 1200 ms cycling frequency slow speed), anti-phase coordination mode at 750 ms cycling frequency (fast speed), anti-phase coordination mode at 1200 ms cycling frequency (slow speed). For the in-phase coordination mode at 750 ms cycling frequency (fast speed) and 1200 ms cycling frequency (slow speed), participants were instructed to reach a start target (depicted white targets appearing at the center of the screen). Once participants reached those start targets, two flashing red targets appeared above and below the white targets. Those red targets flashed at either 750 ms or 1200 ms cycling frequencies, indicating the speed in which the hands had to move. Once the red targets finished flashing, green flashing targets would appear, and participants moved both their hands simultaneously in the same direction during the in-phase movement until the green targets finished flashing. The visual targets were utilized so that participants maintained constant movement amplitude and the distance between the visual targets during oscillations was ten centimeters.

Participants were not informed of the specifics of the perturbations involved in the experiment, rather they were encouraged to continue performing the movements to the best of their ability despite perturbations applied during the experiment. Pauses occurred between each trial, and after a 500 ms delay, the white targets would appear again, and participants reached for those targets to continue the study. For each trial, there were 30 oscillations of the in-phase movement that were performed by participants.

Similarly, in the anti-phase coordination mode at 750 ms cycling frequency (fast speed) and 1200 ms cycling frequency (slow speed), participants were instructed to reach a start target (depicted white targets appearing on the center of the screen). The flashing red targets appeared as described above, and participants moved both their hands simultaneously in the opposite directions in the anti-phase movement until the green targets finished flashing. Again, participants were not aware of the specifics of the perturbations applied but were encouraged to maintain their movements despite applied perturbations. Pauses occurred between each trial, and after a 500 ms delay, the white targets would appear again, and participants reached for those targets to continue the study. For each trial, there were 30 oscillations of the anti-phase movement that were performed by participants.

### Data processing and analysis

All raw data files containing the hand position data, velocity, and acceleration of each limb of the hand, elbow and shoulder joints were imported into MATLAB (R2020b, The MathWorks, USA) for offline data processing using Kinarm MATLAB scripts and custom MATLAB scripts. Both the mean continuous relative phase (ϕ) and the standard deviation of the continuous relative phase (SD_ϕ_) were calculated. To focus specifically on steady performance within every single trial and to compare fast and slow movement speed, we analyzed the movement data from 12 to 42 s per trial for all study participants in both the in-phase and anti-phase coordination modes. It should be noted that during fast and slow speeds, a different number of oscillations are analyzed for the same 30 s time period. In addition, the 9 load conditions were pooled for analysis under the same categories as those described above: in-phase/750 ms cycling frequency, in-phase/1200 ms cycling frequency, anti-phase/750 ms cycling frequency, anti-phase/1200 ms cycling frequency.

### Continuous relative phase (ϕ) & Variability of continuous relative phase (SD_ϕ_) calculation

Continuous relative phase (ϕ) was derived from the Kinarm position data of both the left and the right hands and was used to quantify and characterize the in-phase and anti-phase coordination modes, along with variability (Kelso 1995). Phase angles for left and the right hands were determined using the Hilbert transform approach described by Lamb and Stöckl (Lamb and Stöckl 2014). This approach involved amplitude-centering the kinematic signal around zero (Eq. 4) and calculating phase angles using the position at time *t, x(t),* and their Hilbert transformation $H\left( t \right) \, = \, H\left( {x\left( t \right)} \right)$.

4 $$x_{{{\text{centered}}}} \left( {t_{i} } \right) = x\left( {t_{i} } \right) - \left( {x\left( t \right)} \right) - \frac{{\left( {x\left( t \right)} \right) - \left( {x\left( t \right)} \right)}}{2}$$

The Hilbert transformation outputs a complex analytical signal, ζ(t), where the *H(t)* of *x(t)* act as imaginary components of the analytical signal that can be defined using the following equation (Eq. 5):

5 $$\zeta \left( t \right) = x\left( t \right) + iH\left( t \right)$$

Based on the calculation of this complex signal, the phase angle at time *ti* can be calculated as the inverse tangent via this equation (Eq. 6):

6 $$\phi \left( {t_{i} } \right) = {\text{arctan}}\left( {\frac{{H\left( {t_{i} } \right)}}{{x\left( {t_{i} } \right)}}} \right)$$

The ϕ between the two signals—right hand (*x*_*1*_(*t*)) & left hand (*x*_*2*_(*t*))—was then computed by subtracting the phase angles of the right and the left hand from each other. For instance, the CRP_ϕ_ for the two signals at time *ti* can be computed using the following equation (Eq. 7) where *H*_*1*_(*t*) and *H*_2_(*t*) refer to the Hilbert transformed signal from of the right and the left hand, respectively:

7 $$CRP_{\phi } = \phi_{1} \left( {t_{i} } \right) - \phi_{2} \left( {t_{i} } \right) = {\text{arctan}}\left( {\frac{{H_{1} \left( {t_{i} } \right)x_{2} \left( {t_{i} } \right) - H_{2} \left( {t_{i} } \right)x_{1} \left( {t_{i} } \right)}}{{x_{1} \left( {t_{i} } \right)x_{2} \left( {t_{i} } \right) + H_{1} \left( {t_{i} } \right)H_{2} \left( {t_{i} } \right)}}} \right)$$

These procedures were repeated for each trial, across both the in-phase and the anti-phase coordination modes, for all study participants. The resulting CRP values were between 0 ° and 180 °, where 0 ° denoted a fully in-phase movement pattern and 180 ° denoted a fully anti-phase movement pattern. To summarize, the *ϕ* of the right and the left hand was utilized to quantify and characterize the two coordination modes, while the standard deviation (SD_Φ_) of the *ϕ* indicated variability among *ϕ*.

### Statistical analyses

All statistical analyses were performed using R (Version 1.3.1093). Linear mixed-effects (LME) regression models were fitted using the lme4 package (Bates et al. 2015). To analyze the *ϕ* and SD_ϕ_, we utilized LME models which explicitly accounted for the variation in our data contributed to by each load condition and participant (Gałecki and Burzykowski 2013). Corrections for multiple comparisons were calculated using the Tukey’s method. Estimated marginal means and pairwise comparisons with associated confidence intervals were extracted from the linear regression and computed for each load condition in both the in-phase and anti-phase coordination modes using the emmeans R package (Lenth 2022). The tabular results can be seen in Supplementary Table 1 and the plots showing pairwise comparisons can be seen in Supplementary Fig. 3. We also fitted a linear mixed-effects model to account for the main effect of handedness in our data but ultimately did not find an effect of handedness. Finally, we fitted an LME model to determine if there were any learning effects from one trial to the next in both coordination modes, and results indicated no learning effects.

## Results

### Continuous relative phase (ϕ)

**Fig. 2:**
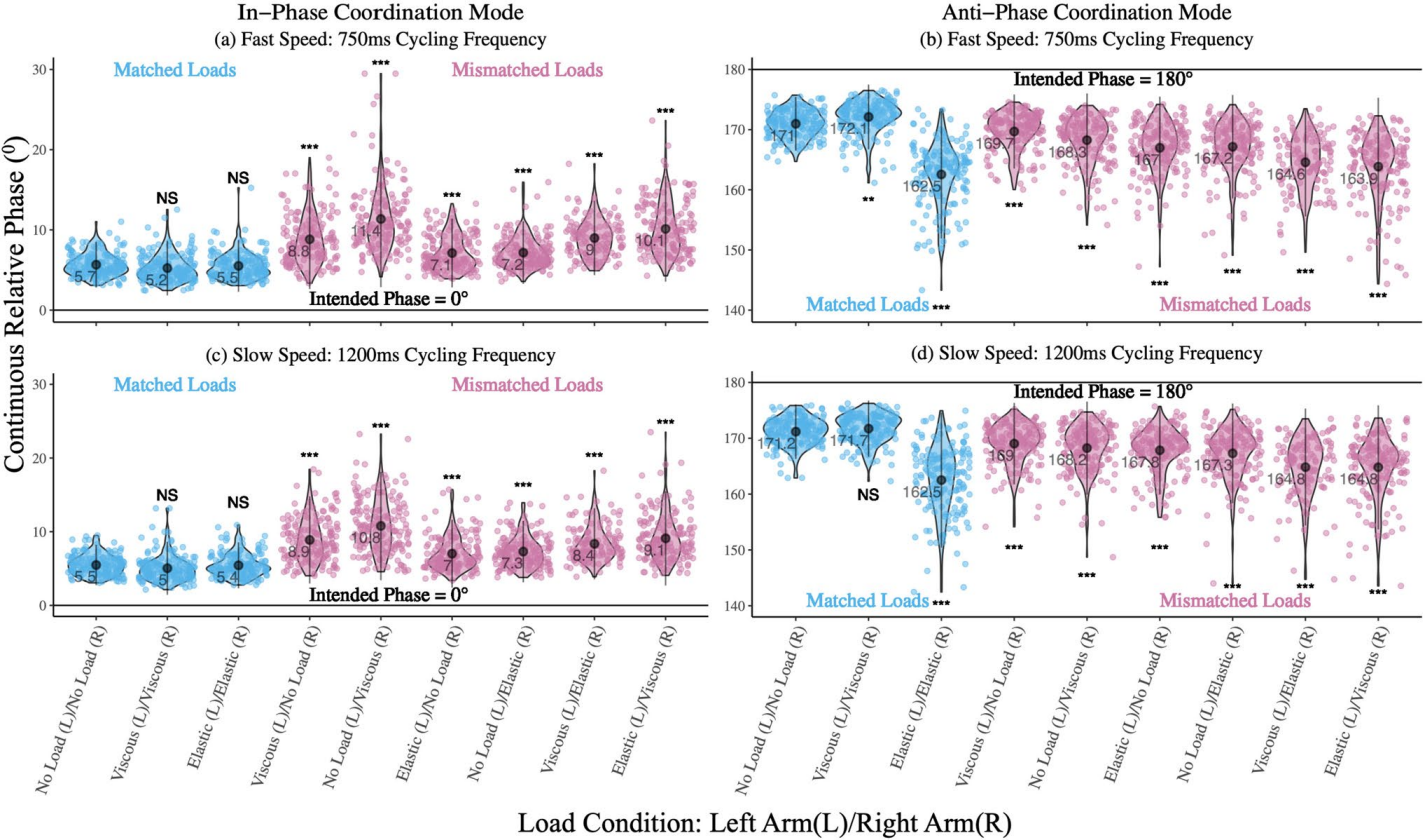
Deviation of Mean Continuous Relative Phase (*ϕ*) from Intended Phase. Visualization of slow and fast oscillation frequencies in mean continuous relative phase during in-phase and anti-phase coordination modes across matched and mismatched load conditions. During in-phase coordination mode, (**a**) and (**c**), the cycling frequencies are closer to 0^0^, whereas during anti-phase coordination mode, (**b**) and (**d**), the cycling frequencies are closer to 180°

Figure 2 shows the deviation of mean continuous relative phase from intended phase during the in-phase (Fig. 2a and c) and anti-phase coordination modes (Fig. 2b and d) at 750 ms and 1200 ms cycling frequencies, respectively. These plots visualize the means of characteristic movement patterns across the different load conditions. Figure 2 displays the phase deviation for matched (where load conditions are same for both hands) vs. mismatched (where load conditions are different for each hand). As can be seen in Fig. 2a, during the in-phase movement at 750 ms cycling frequency, when load conditions are matched (no load/no load: mean 5.7/sd 1.4, viscous/viscous: mean 5.2/sd 1.7, elastic/elastic: mean 5.5/sd 1.6), participants tend to more closely synchronize their movements, i.e., the relative phase is closer to the intended relative phase of 0^0^, compared to when load conditions are mismatched (viscous/no load: mean 8.8/sd 3.1, no load/viscous: mean 11.4/sd 4.2, elastic/no load: mean 7.1/sd 2.1, no load/elastic: mean 7.2/sd 1.9, viscous/elastic: mean 9/sd 2.3 and elastic/viscous: mean 10.1/sd 3.3)—when relative phase is further from the intended relative phase of 0 °. A similar trend is seen in Fig. 2c, during in-phase movement at 1200 ms cycling frequency, where matched (no load/no load: mean 5.5/sd 1.4, viscous/viscous: mean 5/sd 1.8, elastic/elastic: mean 5.4/sd 1.6) load conditions tend to be closer to relative phase of 0 ° compared to mismatched (viscous/no load: mean 8.9/sd 3, no load/viscous: mean 10.8/sd 3.7, elastic/no load: mean 7/sd 2.3, no load/elastic: mean 7.3/sd 2.1, viscous/elastic: mean 8.4/sd 2.4 and elastic/viscous: mean 9.1/sd 3.2) load conditions.

Similarly, during the anti-phase movement at 750 ms cycling frequency (Fig. 2b), matched load conditions (no load/no load: mean 171/sd 2.2, viscous/viscous: mean 172/sd 2.7, elastic/elastic: mean 162.5/sd 5.3) yield lower phase deviation i.e., relative phase is closer to the intended relative phase of 180 °, with the exception of the elastic/elastic matched load condition. Mismatched load conditions (viscous/no load: mean 169.7/sd 3.1, no load/viscous: mean 168.3/sd 3.9, elastic/no load: mean 167/sd 4.2, no load/elastic: mean 167.2/sd 4.3, viscous/elastic: mean 164.3/sd 5.4 and elastic/viscous: mean 163.2/sd 7) at the 750 ms cycling frequency tended to deviate further from this intended phase. Likewise, during the anti-phase movement at 1200 ms cycling frequency (Fig. 2d), matched (no load/no load: mean 171.2/sd 2.4, viscous/viscous: mean 171.7/sd 2.5) load conditions tend to be closer to the relative phase of 180 °, compared to mismatched (viscous/no load: mean 169/sd 3.6, no load/viscous: mean 168.2/sd 4.2, elastic/no load: mean 167.8/sd 3.9, no load/elastic: mean 167.3/sd 4.5, viscous/elastic: mean 164.3/sd 6.6 and elastic/viscous: mean 164.8/sd 5.6) load conditions, which tend to deviate further from this intended relative phase. However, the intended relative phase during matched elastic/elastic forces (elastic/elastic: mean 162.5/sd 6.1) deviated from this pattern, with a larger deviation from the intended relative phase of 180 ° compared to other matched load conditions. Please refer to the polar plots in Supplementary Fig. 1 to visualize continuous relative phase across varying load conditions.

To quantify the differences between movement patterns, across varying load conditions and cycling frequency, a linear mixed-effects model (*ϕ* ~ Load Condition + (1 + Trial) + (1 + Participant)) was implemented for both in-phase and anti-phase coordination modes, with fixed effects of load conditions and random effects of trials and participants to account for variance in the data. During the in-phase coordination mode at 750 ms cycling frequency, there are statistically significant differences between each mismatched load condition, when compared to the null matched condition (with no loads applied to either arm). There were no significant differences between the matched load conditions, when compared to the null matched condition. The same pattern held true for 1200 ms cycling frequency. Thus, only mismatched loads applied during the in-phase coordination mode, at both fast or slow cycling frequencies, significantly increased phase deviation during in-phase bimanual movement.

During the anti-phase coordination mode at 750 ms cycling frequency, there were statistically significant differences in relative phase between the null matched load condition and each of the other matched conditions. We also observed significant differences in relative phase between the null matched load conditions and each of the mismatched load conditions. In the 1200 ms cycling frequency, we again see significant differences in relative phase between the null matched load condition and each mismatched load condition. However, in the matched load conditions, we only observe a significant difference in relative phase for the elastic/elastic matched condition compared to the null matched condition, and no significant differences in relative phase for viscous/viscous. Thus, except for matched viscous loads, any load applied during anti-phase movement at fast or slow cycling frequencies leads to significant increase in phase deviation from 180 ° when compared with null matched loads. Tables 1 and 2 depict the mean continuous relative phase results of the linear mixed effects model for the in-phase and anti-phase movement patterns at 750 ms and 1200 ms cycling frequencies, respectively.

**Table 1:** Linear mixed-effects model (LME) results for Continuous relative phase (*ϕ*) during the 750 ms cycling frequency (fast speed of movement)

	Mean Cont. Relative Phase (*ϕ*)					
	In-phase Movement: 750 Cycling Frequency			Anti-phase Movement: 750 Cycling Frequency		
Fixed effects L = left arm/R = right arm)	*β*	SE	*p(χ*^*2*^*)*	*β*	SE	*p(χ*^*2*^*)*
Intercept (No Load (L)/No Load (R))	5.66	0.29	< **0.001**	170.98	0.56	< **0.001**
Viscous (L)/Viscous (R)	– 0.41	0.25	0.101	1.14	0.38	**0.03**
Elastic (L)/Elastic (R)	– 0.13	0.25	0.606	– 8.43	0.38	< **0.001**
Viscous (L)/No Load (R)	3.17	0.25	< **0.001**	– 1.30	0.38	**0.001**
No Load (L)/Viscous (R)	5.69	0.25	< **0.001**	– 2.72	0.38	< **0.001**
Elastic (L)/No Load (R)	1.47	0.25	< **0.001**	– 3.99	0.38	< **0.001**
No Load (L)/Elastic (R)	1.51	0.25	< **0.001**	– 3.82	0.38	< **0.001**
Viscous (L)/Elastic (R)	3.24	0.27	< **0.001**	– 6.81	0.42	< **0.001**
Elastic (L)/Viscous (R)	4.47	0.25	< **0.001**	– 7.77	0.38	< **0.001**

Random effects	Groups		SD	Groups		SD
	Trial	Intercept	0.18	Trial	Intercept	0.05
	Participant	Intercept	1.33	Participant	Intercept	2.81
	Residual		2.20	Residual		3.50
	Observations: 1445			Observations: 1445		

Statistically significant effects of varying load conditions are shown, bold indicates statistical significance of *p* < 0.05 after Tukey's multiple comparisons test.

**Table 2:** Linear mixed-effects model (LME) results for Continuous relative phase (ϕ) during the 1200 ms cycling frequency (slow speed of movement)

	Mean Cont. Relative Phase (ϕ)					
	In-phase Movement: 1200 Cycling Frequency			Anti-phase Movement: 1200 Cycling Frequency		
Fixed effects L = left arm/R = right arm)	*β*	SE	*p(χ*^*2*^*)*	*β*	SE	*p(χ*^*2*^*)*
Intercept (No Load (L)/No Load (R))	5.49	0.28	< **0.001**	171.18	0.54	< **0.001**
Viscous (L)/Viscous (R)	– 0.45	0.24	0.067	0.55	0.41	0.183
Elastic (L)/Elastic (R)	– 0.05	0.24	0.838	– 8.67	0.41	< **0.001**
Viscous (L)/No Load (R)	3.40	0.24	< **0.001**	– 2.14	0.41	< 0.001
No Load (L)/Viscous (R)	5.28	0.24	< **0.001**	– 2.94	0.41	< **0.001**
Elastic (L)/No Load (R)	1.54	0.24	< 0.001	– 3.33	0.41	< **0.001**
No Load (L)/Elastic (R)	1.82	0.24	< **0.001**	– 3.89	0.41	< **0.001**
Viscous (L)/Elastic (R)	2.82	0.26	** < 0.001**	– 7.02	0.44	< **0.001**
Elastic (L)/Viscous (R)	3.62	0.24	< **0.001**	– 6.39	0.41	< **0.001**

Random effects	Groups		SD	Groups		SD
	Trial	Intercept	0.18	Trial	Intercept	0.15
	Participant	Intercept	1.29	Participant	Intercept	2.65
	Residual		2.12	Residual		3.65
	Observations: 1445			Observations: 1445		

Statistically significant effects of varying load conditions are shown, bold indicates statistical significance of *p* < 0.05 after Tukey's multiple comparisons test

### Variability of continuous relative phase (SD_ϕ_)

**Fig. 3:**
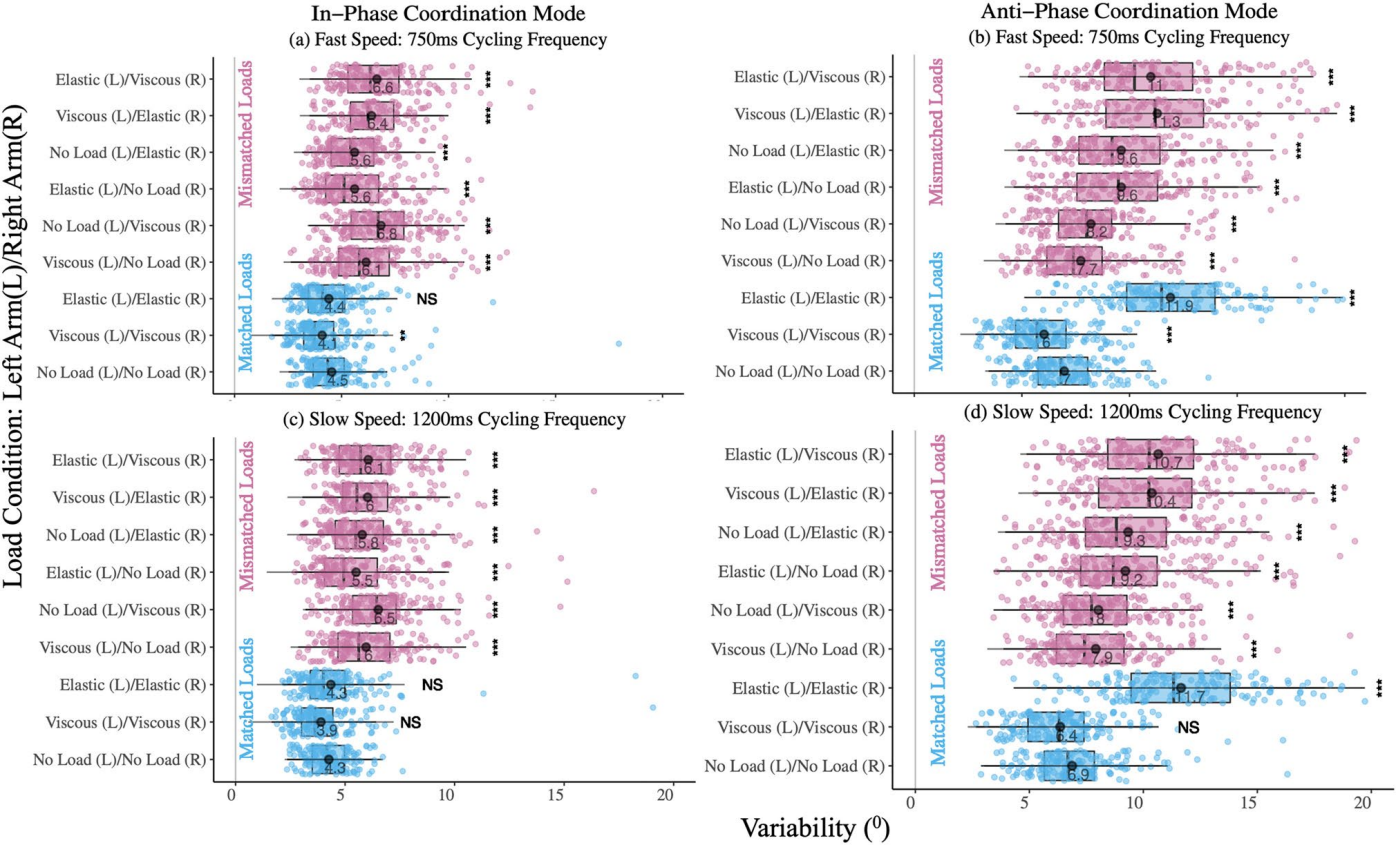
Variability of Mean Continuous Relative Phase (SD_*ϕ*_). Visualizations of slow and fast oscillation frequencies in variability of continuous relative phase, during in-phase and anti-phase coordination modes across matched and mismatched load conditions. Across all coordination modes and cycling frequencies, the mismatched load conditions vary significantly compared to null loads condition. Whereas there are exceptions in the matched load conditions: **a** In-phase fast speed: only the viscous matched loads are significantly variable compared to null loads condition. **b** Anti-phase fast speed: both the elastic and viscous loads vary significantly compared to the null loads. **c** In-phase slow speed: neither the elastic nor the viscous matched loads vary significantly from the null loads condition. **d** Anti-phase slow speed: only the elastic matched loads condition is significantly variable compared to the null loads condition

For variability (SD_*ϕ*_), a linear mixed-effects model (SD_*ϕ*_ ~ Load condition + (1 + Trial) + (1 + Participant)) was used for both the in-phase and anti-phase coordination modes, with the fixed effects of load conditions and random effects of trials and participants to account for variance in the data. In the in-phase coordination mode at 750 ms cycling frequency (Fig. 3a), there were statistically significant differences between each mismatched load condition compared to the null matched load condition. In the matched load conditions, there were significant differences in the viscous/viscous load condition, but not in the elastic/elastic load condition, compared to the null matched load condition. On the other hand, during 1200 ms cycling frequency (Fig. 3c), there were significant differences in variability in all the mismatched load conditions compared to the null matched load condition, and there were no significant differences between the matched load conditions compared to the null matched load condition. Thus, with the exception of matched viscous loads during the 750 ms cycling frequency, any loads applied at the faster speed leads to significant variability in the in-phase movement patterns. Whereas, during the 1200 ms cycling frequency, only mismatched load conditions are significantly variable compared to the null load condition.

In the anti-phase coordination mode at 750 ms cycling frequency (Fig. 3b), we see statistically significant differences in each mismatched and matched load condition compared to the null matched load condition, indicating that at faster speeds the anti-phase movement shows higher variability regardless of whether the loads being applied are matched or mismatched. At the 1200 ms cycling frequency (Fig. 3d), we again see statistically significant differences between each mismatched load condition compared to the null matched load condition, however, only the elastic/elastic matched load condition was significantly different from the null load condition, whereas the viscous/viscous load was not. Based on this data, we can see that when matched elastic/elastic loads are applied to both arms, or when varying forces are applied to either arm leads to greater variability in movement compared to when forces are matched with no loads, or viscous/viscous. Tables 3 and 4 depict the variability results of the linear mixed effects model for the in-phase and anti-phase movement patterns at 750 ms and 1200 ms cycling frequencies, respectively.

**Table 3:** Linear mixed-effects model (LME) results for variability of continuous relative phase (SD_ϕ_) during the 750 ms cycling frequency (fast speed of movement)

Fixed effects L = left arm/R = right arm)	Mean Variability Cont. Relative Phase (SD_ϕ_)					
	In-phase Movement:750 Cycling Frequency			Anti-phase Movement: 750 Cycling Frequency		
	*β*	SE	*p(χ*^*2*^*)*	*β*	SE	*p(χ*^*2*^*)*
Intercept (No Load (L)/No Load (R))	4.54	0.18	< **0.001**	6.97	0.34	< **0.001**
Viscous (L)/Viscous (R)	– 0.45	0.16	**0.004**	– 0.94	0.27	< **0.001**
Elastic (L)/Elastic (R)	– 0.13	0.16	0.400	4.99	0.27	< **0.001**
Viscous (L)/No Load (R)	1.60	0.16	< **0.001**	0.92	0.27	**0.001**
No Load (L)/Viscous (R)	2.30	0.16	< **0.001**	1.23	0.27	< **0.001**
Elastic (L)/No Load (R)	1.07	0.16	< **0.001**	2.65	0.27	< **0.001**
No Load (L)/Elastic (R)	1.07	0.16	< **0.001**	2.71	0.27	< **0.001**
Viscous (L)/Elastic (R)	1.82	0.17	< **0.001**	4.32	0.29	< **0.001**
Elastic (L)/Viscous (R)	2.11	0.16	< **0.001**	4.44	0.27	< **0.001**

Random effects	Groups		SD	Groups		SD
	Trial	Intercept	0.00	Trial	Intercept	0.22
	Participant	Intercept	0.82	Participant	Intercept	1.58
	Residual		1.41	Residual		2.34
	Observations: 1445			Observations: 1445		

Statistically significant effects of varying load conditions are shown, bold indicates statistical significance of *p* < 0.05 after Tukey's multiple comparisons test

**Table 4:** Linear mixed-effects model (LME) results for variability of continuous relative phase (SD_ϕ_) during the 1200 ms cycling frequency (fast speed of movement)

Fixed effects (L = left arm/R = right arm)	Mean Variability Cont. Relative Phase (SD_ϕ_)					
	In-phase Movement: 1200 cycling frequency			Anti-phase Movement:1200 cycling frequency		
	*β*	SE	*p(χ*^*2*^*)*	*β*	SE	*p(χ*^*2*^*)*
Intercept (No Load (L)/No Load (R))	4.26	0.19	< **0.001**	6.88	0.34	< **0.001**
Viscous (L)/Viscous (R)	– 0.24	0.17	0.158	– 0.52	0.28	0.060
Elastic (L)/Elastic (R)	0.09	0.17	0.618	5.00	0.28	< **0.001**
Viscous (L)/No Load (R)	1.70	0.17	< **0.001**	1.04	0.28	** < 0.001**
No Load (L)/Viscous (R)	2.25	0.17	< **0.001**	1.15	0.28	< **0.001**
Elastic (L)/No Load (R)	1.25	0.17	< **0.001**	2.34	0.28	< **0.001**
No Load (L)/Elastic (R)	1.52	0.17	< **0.001**	2.80	0.28	< **0.001**
Viscous (L)/Elastic (R)	1.72	0.18	< **0.001**	4.07	0.30	< **0.001**
Elastic (L)/Viscous (R)	1.80	0.17	< **0.001**	3.85	0.28	< **0.001**

Random effects	Groups		SD	Groups		SD
	Trial	Intercept	0.00	Trial	Intercept	0.21
	Participant	Intercept	0.83	Participant	Intercept	1.60
	Residual		1.56	Residual		2.43
	Observations: 1445			Observations: 1445		

Statistically significant effects of varying load conditions are shown, bold indicates statistical significance of *p* < 0.05 after Tukey's multiple comparisons test

## Discussion

In the current experiment we applied varying force fields—viscous (velocity dependent) and elastic (position dependent)—on the left and the right arms to understand the effect of symmetry breaking (and consequently frequency mismatches between the limbs) on a novel in-phase and anti-phase coordination task. This study builds upon previous in/anti-phase movement tasks (using e.g., pendulums, circle drawing, finger tapping etc., through the novel application for force field perturbations (Sternad et al. 1995; Semjen and Ivry 2001; Spencer et al. 2005; Amazeen et al. 2008; Shih et al. 2019). The present experiment employed a steady-state system of coordination dynamics, whereby we only looked at true in-phase and anti-phase movements without considering any phase transitions that might have occurred, i.e., any trials with phase transitions were excluded during analysis of both the relative phase and variability. Relative phase and variability in relative phase were measured during both the in-phase and anti-phase coordination modes with a total of 9 load conditions: 3 matched load conditions (no load/no load, viscous/viscous, elastic/elastic) and mismatched load conditions (viscous/no load, no load/viscous, elastic/no load, no load/elastic, viscous/elastic and elastic/viscous). As predicted, the anti-phase coordination mode was less stable than the in-phase coordination mode in terms of both relative phase and variability (increased compared to in-phase movement patterns), regardless of matched vs. mismatched conditions. This is consistent with previous research findings where the in-phase movement patterns are more stable, as in-phase movements recruit homologous muscle groups and can be more accurately and effortlessly performed (Haken et al. 1985; Amazeen et al. 1995; Serrien and Brown 2002).

During the in-phase coordination mode for both the 750 ms and 1200 ms cycling frequencies, the relative phase of all mismatched load conditions was significantly different from the matched load conditions. This indicates that during mismatched conditions, when varying loads are being applied to each arm, participants tended to deviate more from the intended phase (Fig. 2a and c) and showed increased variability (Fig. 3a and c), suggesting that varying load conditions created an eigenfrequency difference in the movement of each hand. By applying the elastic force field to one hand and the null force field to the opposite hand, we see an average phase deviation of ~ 7.2 ° (regardless of whether the elastic force field is applied to the left or the right arm). On the other hand, applying the viscous force field to one hand and the null force field to the opposite hand, we see an increase of ~ 10.1 ° in the average phase deviation. Prior research has indicated that when a viscous force field is applied during bimanual rhythmic finger tapping (Mechsner and Knoblich 2004), or unimanual rhythmic wrist flexion and extension (Mackey et al. 2002), participants exhibit a greater production of force. Increasing the force production necessary to move under viscous forces can potentially explain why moving in the same direction (i.e., in-phase) with viscous force field applied to one arm and null force field to the other can result in increased deviation from the intended relative phase.

Similarly, during the anti-phase coordination mode, for both the 750 and 1200 ms cycling frequencies, the relative phase of mismatched load conditions was significantly different from the matched load conditions (except for the viscous/viscous matched loads in the 1200 ms cycling frequency). This indicates that there is a conflict between the spatial (moving when varying loads are applied to each arm, which affects the amplitude of movement, especially in the anti-phase direction), and muscular (where moving synchronously to the flashing targets is easier in the in-phase than the anti-phase direction) task requirements in mismatched load conditions, which affects coordination stability during anti-phase movement (Swinnen et al. 1998, 2001; Carson et al. 2000; Swinnen and Wenderoth 2004). Previously, Park et al. 2001 showed that when detuning is involved, the attractor position (∆*ϕ*) depended on spatial constraints, while the attractor strength (SD_*ϕ*_) depended on muscular constraints. While this effect is not robust during other coordination tasks, i.e., between-person and in-person bimanual coordination (Temprado et al. 2003), it is plausible that spatial constraints affected deviation from relative phase by producing a more pronounced afferent signal to calculate the phase deviation resulting from the movement of the left and the right arms. However, one of the limitations of the HKB model is its inability to capture these spatial and muscular constraints, thus these cannot be fully captured for this task (Peper et al. 2004).

Interestingly, in the anti-phase coordination mode matched load conditions, we see that the elastic/elastic load condition tended to deviate from the intended phase the most (Fig. 2b & d). One possible explanation for this is that the anti-phase movement requires suppression (of a mirrored movement) and the independence to move each arm in the opposite direction. Thus, when loads are being applied, specifically the elastic/elastic load, where the participant’s arm is countering a rubber band-like resistance with the elastic force, it’s creating an interference typically observed during asymmetric bimanual movements, which could be a result of neural crosstalk that arises from the ipsilateral descending pathways (Kennerley et al. 2002). In a model created by Cattaert et al. (1999) each effector receives signals from both the ipsilateral and contralateral descending pathway. The symmetric movement requires activation of the homologous muscle groups and signals of these two pathways are congruent, while the asymmetric movement requires activation of the non-homologous muscle groups, where conflict between the crossed and uncrossed corticospinal pathways may arise, especially when a load is added, as is the case with the elastic/elastic anti-phase movement pattern. Furthermore, from an anatomical standpoint, there are various neural levels where the spatiotemporal coupling of coordination may occur. Motor commands from the primary motor cortex (M1) by way of the corticospinal tract can either take a direct or indirect route to the spinal cord. A majority, approximately 90%, of the axons cross over in the medulla (via the pyramidal decussation) to the contralateral side and terminate on the ventral horn of the spinal cord, forming the lateral corticospinal tract. A small minority, approximately 10%, of the of the axons do not cross at the medulla and run uncrossed through the brainstem to terminate in the medial regions of the spinal cord, constituting the anterior corticospinal tract. These axons of the anterior tract may then terminate contralaterally or ipsilaterally (Swinnen 2002; Carson 2005; Spencer et al. 2005; Welniarz et al. 2017; Calvert and Carson 2022). It is possible that through movement of the contralateral limb, corollary motor commands are also sent from M1 to the ipsilateral limb. Indeed, this is one explanation for the recovery of ipsilesional motor function in patients with upper limb paresis (Calvert and Carson 2022). In this study, during anti-phase movement, each limb may receive conflicting motor commands from each hemisphere of the brain from these descending contralateral and ipsilateral projections. That is, each limb is receiving primarily contralateral motor commands, but also ipsilateral motor commands, due to the non-homologous movement of each limb in anti-phase movement. This may lead to an increase in phase deviation when both hands move in the opposite directions. Prior research has indicated that coupling of proximal muscle groups is stronger than distal muscle groups. In particular, for movement between non-homologous limbs, coordination of isofunctional muscle groups produces more stable coordination than non-isofunctional muscle groups (Meesen et al. 2006). In this study, during anti-phase movement in the elastic/elastic condition, each limb is pushing or pulling (opposite the other) against a potentially different amount of force based on the position of each limb in relation to the position-dependent elastic force field. This may also contribute to increases in phase deviation for anti-phase movement.

We further explored variability of relative phase, which provided us insight into the effect varying load conditions had on movement during the in-phase and anti-phase coordination modes. 0 ° shown in the plots in Fig. 3 indicates perfect in-phase or anti-phase movement. Thus, the closer the data is to 0 °, the less variable the movement. As can be seen in Fig. 3a, every single load condition (matched and mismatched) during 750 ms cycling frequency was significantly variable, apart from the elastic/elastic load condition. This shows that movement with the matched elastic load was easier to sustain. Similarly, in Fig. 3c, during the 1200 ms cycling frequency, every mismatched load condition is significantly variable, but that is not the case of the matched load conditions. Because the participants are moving slower during this in-phase movement, the matched load condition is easier to maintain and is thus, less variable. Next, in Fig. 3b, during the anti-phase movement at 750 ms cycling frequency, we see that both the matched and mismatched load conditions vary significantly, indicating that performing fast anti-phase movement creates higher variability. Similarly, during the anti-phase movement at 1200 ms cycling frequency, all the matched and mismatched loads are highly variable, except for the viscous/viscous load, suggesting that at slower frequency, the viscous load is easier to sustain. Finally, participants seemed to have the most trouble coordinating when the elastic load was present during anti-phase movement, regardless of whether it was in conjunction with the viscous load or with the null load. This suggests that when elastic load was applied to either arm, a higher rate of force was required to compensate for each load condition consisting of the elastic load. Based on this, when elastic load was involved in one or both arms, it created unstable coordination patterns and increased variability overall.

To explore the effect of symmetry breaking by applying varying load conditions in the present study, we minimized learning effects by adopting a randomized design. Load conditions and cycling frequencies were randomized across trials and coordination patterns (in-phase/anti-phase) were presented in separate blocks. To ensure that there were no learning effects, we implemented a linear mixed-effects model to look for learning effects across trials. We found that there were no learning effects in either the in-phase or anti-phase coordination mode in both 750 ms and 1200 ms cycling frequencies, indicating that even though the movement patterns were repetitive, this did not translate to learning and improving over the course of the experiment. Please see Supplementary Fig. 2 for plots visualizing the learning effects.

Finally, there are three ways in which the varying load conditions can potentially explain movement patterns in the in-phase and anti-phase coordination modes: (1) the cycling frequency and the strength of the motor command can potentially compensate for the load conditions, (2) the strength of the arms muscles varies within participants and could have made a difference during the varying load conditions, and (3) the use of information to correct movement errors could have influenced the movement patterns, and affected the results seen here. All in all, this study provides another method of studying bimanual coordination in varying load conditions. These varying conditions provide a way of investigating neural coupling feedback strategies, and neuromuscular constraints and their effect on continuous relative phase and variability of movement patterns. Furthermore, this work has implications for rehabilitation, specifically for hemiparetic stroke rehabilitation. Hemiparetic stroke gives rise to abnormalities of muscle tone, i.e., spasticity, weakness in muscles and interference with coordination. The primary source of movement dysfunction in these patients is due to abnormal movement coordination (Dewald et al. 2001). Given that bimanual coordination involves multiple brain areas by way of the corpus collosum, post-stroke patients tend to show deficits in bimanual task performance. Bilateral training coupled with applying load to the non-paretic arm has previously been shown to improve task performance, as simultaneous activation of bilateral movements activates a balance in interhemispheric interaction, inducing neural coupling (Cardoso de Oliveira et al. 2001; Cauraugh et al. 2009; Sleimen-Malkoun et al. 2011). Thus, by either increasing the movement time or applying a load to the non-paretic limb, while keeping constant the difficulty of the stroke affected arm, may make bilateral movements more symmetric by increasing the effect of neural coupling and improving synchronization overall, resulting in improvement in upper limb coordination between limbs.

## Conclusion

Performing bimanual coordination with both arms in varying load conditions affected both the continuous relative phase (*ϕ*) and variability (SD_*ϕ*_) of movement patterns in both the in-phase and anti-phase coordination modes. When the load conditions were matched, the *ϕ* was close to the intended relative phase (0 ° for in-phase movement patterns and 180 ° for anti-phase movement patterns) and SD_*ϕ*_ was lower, compared to mismatched load conditions. The effect of applying viscous and elastic force fields to both arms simultaneously produced greater phase deviation and higher variability compared to when there was only one load on one arm and no load on another arm. The only exception to this result was the elastic/elastic load condition in the anti-phase coordination mode, which had the highest *ϕ* and SD_*ϕ*_. In this scenario it is plausible as the elastic force is resistive from the starting position and increases in response to an increase in movement, in addition to the fact that performing anti-phase movement requires more attentional resources than performing in-phase movement. In sum, during the in-phase and anti-phase coordination mode, both the mismatched load conditions show an increase in continuous relative phase and variability compared to the matched load conditions, with in-phase movement being more stable compared to anti-phase movement patterns.

## Supplementary Information

Below is the link to the electronic supplementary material.

Supplementary file1 (DOCX 1785 KB)

## Data Availability

Datasets generated and analyzed during the current study are available from the corresponding author upon reasonable request.
