# Supplementary material for: The effect of elastic and viscous force fields on bimanual coordination
Source: Exp Brain Res. 2023 Mar 14;241(4):1117–30. doi: 10.1007/s00221-023-06589-7 (PMC10081978; doi:10.1007/s00221-023-06589-7)
Supplement: Supplementary file 1 — Supplementary file1 (DOCX 1785 KB) [file 221_2023_6589_MOESM1_ESM.docx]

**Supplementary Information**

**The Effect of Force Field Detuning on Bimanual Coordination**

**Jaskanwaljeet Kaur^1,*^, Shannon Proksch^1,2^, Ramesh Balasubramaniam^1^**

^1^University of California-Merced, Cognitive and Information Sciences, Merced, California, USA

^2^Augustana University, Psychology Department, Sioux Falls, South Dakota, USA

^*^jkaur28@ucmerced.edu

Supplementary Figure 1.

**S1.** Polar plots depicting limb movement in the in-phase and anti-phase coordination modes. (a) & (c) refer to the mean continuous relative phase of all participants during the in-phase coordination mode at 750ms and 1200ms cycling frequencies, respectively. (b) & (d) refer to the mean continuous relative phase of all participants during the anti-phase coordination mode at 750ms and 1200ms cycling frequencies, respectively. The length of the colored lines in the plots refer to variability, where the longer lines denote greater variability compared to a shorter line. As can be seen here, the in-phase coordination mode centers around 0^0^, while the anti-phase coordination mode centers around 180^0^, with variability in relative phase seen during varying load conditions. Values around the polar plot range from 0^0^ to 360^0^.

Supplementary Figure 2.

**S2.** Serial plots depicting the relative phase behavior as varying load conditions are applied over time. Shown are (a) the 750ms cycling frequency, in-phase coordination mode, (b) the 1200ms cycling frequency, in-phase coordination mode, (c) the 750ms cycling frequency, anti-phase coordination mode and (d) the 1200ms cycling frequency, anti-phase coordination mode. All plots are showing trials averaged across all 33 participants. As can be seen there are no learning effects seen overtime, as participants performed in-phase and anti-phase movements.

Supplementary Table 1: Estimated Marginal Means.

| **In-Phase Coordination Mode**  **Fast Speed: 750ms Cycling Frequency** | | | | |
| --- | --- | --- | --- | --- |
| **Loads Applied**  **Left Arm/Right Arm** | **emmean** | **SE** | **lower.CL** | **upper.CL** |
| No Load/No Load | 5.66 | 0.30 | 5.06 | 6.25 |
| Viscous/Viscous | 5.24 | 0.29 | 4.66 | 5.82 |
| Elastic/Elastic | 5.53 | 0.29 | 4.94 | 6.11 |
| Viscous/No Load | 8.83 | 0.29 | 8.24 | 9.41 |
| No Load/Viscous | 11.35 | 0.29 | 10.77 | 11.93 |
| Elastic/No Load | 7.13 | 0.29 | 6.55 | 7.71 |
| No Load/Elastic | 7.16 | 0.29 | 6.58 | 7.74 |
| Viscous/Elastic | 8.90 | 0.31 | 8.29 | 9.51 |
| Elastic/Viscous | 10.12 | 0.29 | 9.54 | 10.70 |
| Confidence level used: 0.95 | | | | |

| **In-Phase Coordination Mode**  **Slow Speed: 1200ms Cycling Frequency** | | | | |
| --- | --- | --- | --- | --- |
| **Loads Applied**  **Left Arm/Right Arm** | **emmean** | **SE** | **lower.CL** | **upper.CL** |
| No Load/No Load | 5.49 | 0.29 | 4.91 | 6.06 |
| Viscous/Viscous | 5.04 | 0.28 | 4.48 | 5.60 |
| Elastic/Elastic | 5.44 | 0.28 | 4.87 | 6.00 |
| Viscous/No Load | 8.89 | 0.28 | 8.33 | 9.46 |
| No Load/Viscous | 10.77 | 0.28 | 10.20 | 11.33 |
| Elastic/No Load | 7.03 | 0.28 | 6.46 | 7.59 |
| No Load/Elastic | 7.31 | 0.28 | 6.74 | 7.87 |
| Viscous/Elastic | 8.31 | 0.30 | 7.72 | 8.90 |
| Elastic/Viscous | 9.10 | 0.28 | 8.54 | 9.67 |
| Confidence level used: 0.95 | | | | |

| **Anti-Phase Coordination Mode**  **Fast Speed: 750ms Cycling Frequency** | | | | |
| --- | --- | --- | --- | --- |
| **Loads Applied**  **Left Arm/Right Arm** | **emmean** | **SE** | **lower.CL** | **upper.CL** |
| No Load/No Load | 171 | 0.56 | 170 | 172 |
| Viscous/Viscous | 172 | 0.56 | 171 | 173 |
| Elastic/Elastic | 163 | 0.56 | 161 | 164 |
| Viscous/No Load | 170 | 0.56 | 169 | 171 |
| No Load/Viscous | 168 | 0.56 | 167 | 169 |
| Elastic/No Load | 167 | 0.56 | 166 | 168 |
| No Load/Elastic | 167 | 0.56 | 166 | 168 |
| Viscous/Elastic | 164 | 0.58 | 163 | 165 |
| Elastic/Viscous | 163 | 0.56 | 163 | 164 |
| Confidence level used: 0.95 | | | | |

| **Anti-Phase Coordination Mode**  **Slow Speed: 1200ms Cycling Frequency** | | | | |
| --- | --- | --- | --- | --- |
| **Loads Applied**  **Left Arm/Right Arm** | **emmean** | **SE** | **lower.CL** | **upper.CL** |
| No Load/No Load | 171 | 0.55 | 170 | 172 |
| Viscous/Viscous | 172 | 0.55 | 171 | 173 |
| Elastic/Elastic | 163 | 0.55 | 161 | 164 |
| Viscous/No Load | 169 | 0.55 | 168 | 170 |
| No Load/Viscous | 168 | 0.55 | 167 | 169 |
| Elastic/No Load | 168 | 0.55 | 167 | 169 |
| No Load/Elastic | 167 | 0.55 | 166 | 168 |
| Viscous/Elastic | 164 | 0.58 | 163 | 165 |
| Elastic/Viscous | 165 | 0.55 | 164 | 166 |
| Confidence level used: 0.95 | | | | |

Supplementary Figure 3.

**S3.** Pairwise Comparisons of Estimated Marginal Means across different movement speeds and coordination modes: (a) In-phase coordination mode/Fast speed at 750ms Cycling Frequency, (b) Anti-phase coordination mode/Fast speed at 750ms Cycling Frequency, (c) In-phase coordination mode/Slow speed at 1200ms Cycling Frequency, and (d) Anti-phase coordination mode/Slow speed at 1200ms Cycling Frequency. Blue bars in the plots represent 95% confidence intervals. Red arrows represent comparison among means. When the red arrows overlap with a red arrow from another category, the difference between the overlapping categories is not significant.
